# Mess. Tatham and Egg's Newly Invented Truss

**Published:** 1801-09

**Authors:** 

**Affiliations:** Charing Cross; Charing Cross


To the EDITORS of the MEDICAL and PHYSICAL JOURNAL.

Gentlemen,

WE beg leave to inform you of a newly invented Truss, of which we are the patentees, and which we are assured, by several professional gentlemen of the first respectability, far exceeds any other which has hitherto been invented, both for durability and effect. Its spring being much stronger and more highly tempered than any other, it is capable of being more exactly fitted to the parts on which it is to act, and of resisting any force or exertion by which it might be liable to be misplaced; nor is its influence to be weakened by long use.

The annexed letter we received from the surgeon of the Greenwich Hospital, and we have since had the honour of being appointed by the honourable board to supply that institution. We have also the appointment to the Chelsea Hospital, beside other appointments to workhouses and charities, and also the universal approbation of every individual who has been recommended to us by the Faculty. We are persuaded the more this invention is known, the more it will be prefered for its peculiar advantages over every other hitherto offered to the public.

We are, &c.

*Charing Cross, Aug.* 19, 1801. TATHAM and EGG.

P. S. Sold only at Tatham and Egg's, gun makers, No. 37, Charing Cross.

---

Gentlemen, *Royal Infirmary, June* 24, 1801.

AGREEABLY to your request I herewith send you the result of my trial, of two months, of your elastic trusses; their durability and security are certainly superior to any thing of the kind, I have ever met with.

On

On the first application of them, a tightness is complained of, common to all trusses, but they grow easier by use, and I do not find that straps are absolutely necessary to them; and, therefore, it is my opinion, that when the consideration of the above qualities is added to that of their very moderate price, it must be an object to any chirurgical institution to give them the preference. I am, &c.

W. MILLER.

To Meſſrs. Tatham and Egg, Gun Makers,
No. 37, Charing Cross, London.

